# Acute tubulointerstitial nephritis (ATIN) including drug-induced, tubulointerstitial nephritis and uveitis (TINU), ANCA-associated vasculitis (AAV), kidney- limited sarcoidosis, and hemolysis: a case series from Syria

**DOI:** 10.1186/s12882-025-04030-5

**Published:** 2025-02-25

**Authors:** Mohammad Alsultan, Marwa Kliea, Qussai Hassan

**Affiliations:** 1 Department of Nephrology, Damascus University- Faculty of Medicine, Damascus, Syria; 2 Department of Neurology, Damascus University- Faculty of Medicine, Damascus, Syria; 3 Nephrology Department, Damascus University- Faculty of Medicine, Omar Ibn Abdulaziz Street, Al Mazah, Damascus, Syria

**Keywords:** Acute tubulointerstitial nephritis (ATIN), Drug-induced ATIN (DI-ATIN), Tubulointerstitial nephritis and uveitis (TINU) syndrome, ANCA-associated vasculitis (AAV), Hemolysis

## Abstract

**Background:**

This study aimed to detail acute tubulointerstitial nephritis (ATIN) patients, from relevant clinical manifestations to outcomes.

**Methods:**

We reviewed ATIN patients between 2018 and 2022. All demographic data, labs, biopsy findings, treatment protocols, and outcomes were reported.

**Results:**

ATIN was diagnosed in nine patients, eight by kidney biopsy and one clinically. Drug-induced ATIN (DI-ATIN) was reported in five patients, including rifampin (RIF), allopurinol, mesalamine, and two with cephalosporins. Severe ATIN resulted after the first dose of RIF aligned with liver injury, hemolysis, and thrombocytopenia. Also, mesalamine and allopurinol induced gradual kidney failure a few months after the drug initiation. A patient with Tubulointerstitial nephritis and uveitis (TINU) syndrome showed refractory uveitis presenting during glucocorticoids (GCs) tapering, which resolved quickly with azathioprine (AZA) when not responding to GCs reescalation. Among the rarest cases, ATIN induced by a kidney-limited sarcoidosis, G6PD patient with hemolysis induced ATIN, and isolated ATIN induced by ANCA-associated vasculitis (AAV) with positive C-ANCA, which the latter representing the first case in our country and the fourth case worldwide. Labs showed anemia (88.8%), ESR elevation (85.7%), microscopic hematuria (in all patients), pyuria (44.4%), and proteinuria (77.7%). Biopsies showed interstitial infiltrations mainly with lymphocytes and monocytes. Eosinophils were found in one biopsy and neutrophils showed in 4 biopsies (50%).

**Conclusion:**

ATIN is a disease with a diagnostic challenge, thus clinicians should maintain a high suspicion for diagnosis. The combination of AKI with positive tests (especially abnormal urine sediment, ESR elevation, and anemia) may suggest ATIN diagnosis and further support the treatment initiation, particularly when kidney biopsy is unable to be performed or when the inciting agent is predictable.

## Introduction

Acute tubulointerstitial nephritis (ATIN) is an inflammatory disease characterized by infiltration of inflammatory cells in the kidney interstitium [1]. The exact incidence of ATIN is difficult to define mostly due to differences in policies to perform kidney biopsies worldwide [2]. ATIN is reported in 2–3% of kidney biopsies; however, the prevalence is increased in kidney biopsies for diagnosis of acute kidney injury (AKI) patients to be as high as 13–25% [3, 4].

ATIN has multiple etiologies, including drug-induced, infectious, autoimmune, and idiopathic, the latter comprising 8% of cases [3, 5]. Drug-induced ATIN (DI-ATIN) is the most common etiology, which is responsible for almost two-thirds of the cases (approximately 70%) [3, 4]. More than 250 drugs have been reportedly associated with ATIN; however, antibiotics, proton pump inhibitors (PPIs), and nonsteroidal anti-inflammatory drugs (NSAIDs) consist of the most frequently implicated drugs, specifically each of antibiotics and NSAIDs represent 35% of DI-ATIN cases [3, 4]. In fact, almost any drug could cause ATIN and should be considered a suspected causative agent [2].

DI-ATIN is characterized by several important features, that every nephrologist should know. Firstly; only a small percentage of individuals develop DI-ATIN often associated with systemic hypersensitivity manifestations [6]. Secondly, it is a dose-independent type IV hypersensitivity reaction that typically occurs in kidney parenchyma 7–10 days after drug exposure [3, 6]. Thirdly, it may recur after re-exposure to the same drug or a drug from the same class [6].

The second most common etiology of ATIN is autoimmune disorders, which account for up to 5–20% of biopsy-proven ATIN [2]. Although ATIN mostly occurs in response to glomerular injury, it may also occur in isolation [2]. Multiple autoimmune diseases have been associated with ATIN, such as sarcoidosis, Sjogren’s syndrome, tubulointerstitial nephritis and uveitis (TINU) syndrome, and ANCA-associated vasculitis (AAV) [2, 4].

Infections are a less common cause of ATIN, accounting for 4–17% of cases [2]. Several pathogens have been implicated, including bacterial (such as Brucella and Legionella), viral (such as cytomegalovirus and Epstein–Barr), and mycobacterium (such as tuberculosis (TB) and mycoplasma) [2]. Also, bacterial pyelonephritis could be associated with ATIN and should be suspected when interstitial infiltrates predominantly consist of neutrophils [1, 7].

Previous data suggest that both cell-mediated and antibody-mediated immunities are involved in the ATIN pathogenesis [6]. ATIN is a hypersensitivity reaction that is directed against a renal antigen or mostly induced by extrarenal antigens, particularly drugs or infectious agents, that bind to kidney structures [6]. In fact, cell-mediated immunity seems to play a major role in ATIN, where numerous T cells usually infiltrate the interstitium and may form granulomas [6].

The great challenge in ATIN is particularly due to non-specific manifestations [7]. The delayed diagnosis is usually multifactorial, attributed to non-specific symptoms, no definitive noninvasive tests, and the presence of a latent period between the inciting agent and clinical presentation, which may occur within days or even after more than a year [6]. Specifically; NSAIDs and PPIs might cause ATIN after several months of treatment initiation with minimal or no clinical symptoms at all [6]. Thus, combined with all these points, ATIN diagnosis seems challenging and should be considered in any patient with unexplained AKI [2].

Kidney dysfunction typically presents with a non-oliguric AKI; however, it may be severe and dialysis is required in about one-third of patients [6, 7]. Numerous extrarenal symptoms corresponding with hypersensitivity reaction have been reported, including low-grade fever, rash, arthralgias, flank pain, anorexia, nausea/ vomiting (N/V), malaise, lymphadenopathy, and eosinophilia [6, 7]. The combination of the classic triad in ATIN; which includes fever, eosinophilia, and rash, was reported in < 10% of patients [6, 7].

In this study, we reviewed the first case series of ATIN in Syria to enrich the medical literature for such disease. This study aimed to detail ATIN, from relevant clinical manifestations to features of kidney biopsies and treatment protocols aligned with long-term outcomes.

## Methods

We retrospectively reviewed the medical records of ATIN patients in the Nephrology department at Al Assad University Hospital between 2018 and 2022. This case series included eight patients with ATIN diagnosed by kidney biopsy, and one patient was diagnosed as probable ATIN by a history of causative agents, symptoms, and laboratory findings. Demographic data (Table 1) included age, gender, medical history, signs and symptoms, presence of uveitis (diagnosed by ophthalmological examination), and the probable etiology of ATIN. Laboratory data (Table 2) included white blood count (WBC), hemoglobin (Hb), urea (Ur), creatinine (Cr), glomerular filtration rate (GFR), albumin (ALB), potassium (K), erythrocyte sedimentation rate (ESR), C- Reactive Protein (CRP), and urinalysis. Also, immunological tests were requested as needed (Table 2), which included complement C3 and C4, antinuclear antibody (ANA), perinuclear and cytoplasmic anti-neutrophil cytoplasmic antibodies (P and C- ANCA, respectively), immunoglobulins (Ig), and light chains. Histopathological findings of the kidney biopsy (Table 3) included the total number of glomeruli aligned with the number of hyalinized glomeruli, interstitial inflammation, interstitial fibrosis, tubular changes, blood vessels findings (i.e., subintimal fibrosis and lumens occlusion), and immunofluorescence. Treatment agents, the treatment duration, and outcomes were also reported.

**Table 1:** Demographic and clinical features

Patient no	Age (y)	Gender	Medical history	Symptoms/ Signs	Uveitis	Cause
1	16	F	Neg	Fever, fatigue, reduced appetite and weight	Pos *	TINU
2	42	F	Pneumonia	Abdominal pain, N/V, polyuria	Neg	Antibiotics for pneumonia
3	45	F	Brucellosis	HA, fever, arthralgia, rash, dyspnea, N/V, diarrhea, abdominal pain, anaphylactic shock, jaundice, anuria	Neg	Rifampicin
4	63	M	HTN	Fatigue, rash, itching	Neg	Allopurinol
5	23	M	UC	Asymptomatic elevated Cr in routine tests	Neg	Mesalamine
6	57	M	G6PD	Fatigue, reduced appetite, N/V, diarrhea, abdominal pain, fever, jaundice, dark urine	Neg	Hemolysis
7	50	M	HTN	Numbness in all limbs, progressive weakness of the lower extremities, arthralgia, hearing loss, livedo reticularis, anuria, edema, pulmonary edema	Neg	AAV
8	43	F	Neg	Fatigue, N/V, flank and loin pain, reduced appetite	Neg	Ceftriaxone
9	55	F	Neg	Reduced appetite and weight, nocturia, fatigue	Neg	Sarcoidosis

* Uveitis appeared two months after nephritis

M; male, F; female, TINU; Tubulointerstitial nephritis and uveitis, N/V; nausea and vomiting, HTN; hypertension, UC; ulcerative colitis, Cr; creatinine, G6PD; Glucose-6-phosphate dehydrogenase deficiency, AAV; ANCA associated vasculitis, ND; not defined

**Table 2:** Initial laboratory findings

Patient no	WBC	Hb	Ur	Cr	GFR	ALB	K	ESR	CRP	Urinalysis	Others
1	6.1	10.7	55	3.3	20	3.9	4.1	90	7.3	WBC = 30, RBC = 5, Prot (+)	Neg (C3, C4, ANA)
2	8	9.7	61	3.3	17	3.6	3.3	99	2.4	WBC = 5, RBC = 5, Prot (+)	Neg (C3, C4, ANA, P-ANCA, C-ANCA)
3	9.7	8.7	187	11	4	3	4	ــــ	2	WBC = 20, RBC = 200, Prot (-)	PLT = 119, T-Bil = 1.4, D-Bil = 1.3, LDH = 725
4	7.4	13.8	179	4	16	4.3	3.7	ــــ	0.1	WBC = 4, RBC = 4, Prot (+)	ــــ
5	8.2	12.2	48	2	47	4	3.7	35	0.3	WBC = 10, RBC = 100, Prot (-), Up/24 h = 360 mg	ــــ
6*	5.7	11.1	289	13.6	4	3.1	4	83	5.5	WBC = 60, RBC = 20, Prot (+), Hb= ++, Up/24 h = 196 mg	Neg (C3, C4, dir-coombs, ind-coombs T-Bil = 2.5, ind-Bil = 1.9
7*	21.9	10.4	259	7.3	8	3.4	4.9	93	ــــ	WBC = 7, RBC = 150, Prot (+)	Neg (C3, C4, P-ANCA) Pos C-ANCA
8	7	9.9	22	3.5	16	3.2	4	55	ــــ	WBC = 5, RBC = 5, Prot (++), Up/24 h = 1 g	Neg (C3, C4, Ig, bence jones, lambda, protein electrophoresis) Pos kappa.
9*	7.7	8.4	129	6.1	8	3.5	4.5	80	0.2	WBC = 5, RBC = 5, Prot (+)	Neg (C3, C4, ANA, ds-DNA, P-ANCA, C-ANCA, Ig, kappa, lambda, bone marrow biopsy, TB tests, chest HRCT)

*Emergent hemodialysis sessions were performed

WBC; white blood count, HB; hemoglobin, Ur; urea, Cr; creatinine, GFR; Glomerular filtration rate, ALB; albumin, K; potassium, ESR; erythrocyte sedimentation rate, CRP; C- Reactive Protein (up to 0.5 mg/L), RBC; red blood cell, Prot; protein, Up/24 h; urinary protein in 24 h, C3; complement C3 (range 90–180), C4; complement C4 (range 10–40), ANA; antinuclear antibody; P- ANCA; perinuclear anti-neutrophil cytoplasmic antibodies, C-ANCA; cytoplasmic ANCA, PLT; platelets, T-Bil; total bilirubin, D-Bil; direct bilirubin, LDH; lactate dehydrogenase, dir-coombs; direct coombs, ind-coombs; indirect coombs, ind-Bil; indirect bilirubin, Ig; immunoglobulins, ds-DNA; anti-double stranded DNA, TB tests; tuberculosis, HRCT; high-resolution computed tomography

**Table 3:** Biopsy findings, treatments, and outcomes

Patient no	Biopsy findings					Treat	Duration	Outcome (Cr/6 m)
	Total glomeruli	Interstitial inflammation^#^	Interstitial fibrosis (% of renal cortex)^€^	Tubules	Others			
1	35	lymph, mono, Neutr	< 10%	ATN + Neutr casts in tubules	ــــ	MP + PD + AZA	> 1y	0.8
2	24	lymph, mono, Neutr,	10% with edema	ATN + Neutr casts in tubules	1 glomerulus is hyalinized + vessels occlusion 25–50% of the lumens	PD	6 m	1.1
3	21	lymph, mono	15%	ATN	ــــ	MP + PD	6 m	0.8
4 ^¥^	ــــ	ــــ	ــــ	ــــ	ــــ	PD	3 m	1.3
5	11	lymph, mono, Neutr	10%	ATN	4 glomeruli are hyalinized + Vessels occlusion 25% of the lumens	PD + AZA	PD for 6 m AZA until now ^£^	1.29
6 ^$^	32	lymph, mono	20%	ATN + chronic tubular changes	5 glomeruli are hyalinized + Vessels occlusion 50% of the lumens	MP + PD	3 m	0.9
7	11	lymph, mono, Neutr	10%	ATN + Neutr casts in tubules	Vessels occlusion 25% of the lumens	MP + PD + CYP	1 y ^α^	1.3 ^α^ Dead
8	18	Lymph, eosin	20%	Tubular atrophy + Hyaline and neutr casts in tubules	1 glomerulus is hyalinized + Vessels occlusion 50% of the lumens	PD	6 m	1.06
9	8	Non-necrotizing granulomas with epithelioid and giant cells	15%	ATN	2 glomeruli are hyalinized + Vessels occlusion 50% of the lumens	PD	1y ^β^	1.7

^**#**^ Neutr; neutrophiles; mono: monocytes, lymph: lymphocytes, eosin: eosinophiles

^€^ Interstitial fibrosis by trichrome stain

^¥^ The biopsy was not performed

^$^ This biopsy showed acute and chronic TIN

^£^ AZA treatment continued until now for UC

^α^ Cr reached 1.3 mg/dL after a month and continued for 6 months, followed by deterioration and died after a year

^β^ the patient still on a maintenance dose of PD

Immunofluorescence in all patients was negative for immunoglobulins, complement, and light chains

ATN; acute tubular necrosis, Cr; serum creatinine, PD; prednisone, MP; intravenous methylprednisolone, AZA; azathioprine, CYP; cyclophosphamide, m; months, y; year

The data has been inserted into Microsoft Excel Worksheet 2019 and expressed as mean ± standard deviation and proportions.

## Results

### General description

The study included 9 patients (Table 1). The sample included 5 females (55.5%) and 4 males (44.4%) with a mean age of 43.7 years. The symptoms ranged from an asymptomatic elevation of serum creatinine (sCr) (the 5th patient) to anaphylactic shock (the 3rd patient). The drugs, that were implicated in ATIN, were reported in 5 patients (55.5%), which included cephalosporines antibiotics (probably cephalosporins) in the 2nd and 8th patients, rifampicin in the 3rd patient, allopurinol in the 4th patient, and mesalamine in the 5th patient.

The labs are shown in Table 2. The mean Hb was 10.54 g/dL, where 8 patients (88.8%) showed anemia. The mean GFR was 15.5 ml/ min/ 1.73m^2^, ranging from 4 to 47 ml/ min/ 1.73m^2^; however, three patients received emergent hemodialysis (HD) sessions during admission. Erythrocyte Sedimentation Rate (ESR) was drawn in 7 patients, where elevated ESR was shown in 6 patients (6/7; 85.7%), and the mean ESR was 76.42 mm/hr. Microscopic hematuria showed in all patients, pyuria showed in 4 patients (44.4%), and proteinuria was showed in 7 patients (77.7%). Immunological tests were negative in all patients except the 7th patient, who showed positive C-ANCA.

Kidney biopsy (Table 3) was applied for 8 patients, the mean glomeruli was 20, and 5 biopsies showed hyalinized glomeruli. The interstitial inflammation mainly showed infiltrations with lymphocytes and monocytes aligned with neutrophils (in 4 patients; 50%), and only the 8th patient showed eosinophils infiltrations. The interstitial fibrosis was mild (< 25% of renal cortex) in all biopsies. The tubules mainly showed ATN in 7 biopsies (7/8; 87.5%) aligned with neutrophil casts (in 4 biopsies; 50%) in tubular lumens. Immunofluorescence was negative for immunoglobulins, complement, and light chains in all biopsies.

The treatment (Table 3) included glucocorticoids (GCs), mainly prednisone (PD) in all patients with intravenous (IV) bolus of methylprednisolone (MP) in 4 patients, Azathioprine (AZA) was prescribed for two patients, and cyclophosphamide (CYP) in one patient. The treatment duration ranged from 3 to 6 months; except for the following patients: the 1st patient continued AZA for more than a year for uveitis, the 5th patient continued AZA until now to treat ulcerative colitis (UC), the 7th patient continued treatment for AAV until he died a year after, and the 9th patient continued a maintenance dose of PD for 3 years for sarcoidosis.

### Specific cases

The 1st patient developed anterior uveitis two months after nephritis and was diagnosed with TINU. AZA was added to this patient to treat uveitis when it developed during PD tapering and showed resistance to PD escalation. The treatment continued for more than a year and follow-up continued for two years with good response to AZA and no further uveitis or nephritis relapses.

The 3rd patient presented with anaphylactic shock after receiving the first dose of rifampicin (RIF) for treating Brucellosis and was admitted to the hospital. On the next day, the patient developed AKI, acute liver injury (elevated liver enzymes and jaundice), hemolysis, and thrombocytopenia. Two days later, acute liver injury was recovered spontaneously. A kidney biopsy showed ATIN, which was recovered by GCs treatment.

The 4th patient presented with gradual kidney failure for four months after receiving allopurinol for hyperuricemia. The diagnosis was made clinically when symptoms developed after allopurinol administration and the patient refused kidney biopsy. Kidney function was recovered partially with three months of PD treatment, where the baseline sCr = 0.9 mg/dL and after ATIN return to sCr = 1.3 mg/dL. The kidney function is stable for two years of follow-up.

The 6th patient with G6PD presented gallstones pancreatitis that induced hemolysis and AKI and received two emergent HD sessions during admission. When kidney function did not recover by conservative management, the kidney biopsy was performed. Kidney biopsy showed ATIN superimposed on chronic changes without RBCs or hyaline casts. The patient received MP followed by PD for 3 months, where kidney function was recovered (Cr = 0.9 mg/dL) after the first month. The follow-up continued for 5 years and no further hemolysis or AKI recurrences.

The 7th patient presented with numbness in all limbs, progressive weakness of the lower extremities (strength 3/5), arthralgia, livedo reticularis, hearing loss, anuria, and edema. The patient was admitted to the intensive care unit (ICU) due to pulmonary edema and received three pulses of MP with four emergent HD. The kidney biopsy showed ATIN with positive C-ANCA, where the diagnosis corresponded with AAV. Thereafter, the patient continued the induction therapy with PD and six pulses of cyclophosphamide (CYP). After a month, the kidney function gradually improved, where sCr returned to 1.3 mg/dL, which continued stable for 6 months. Neuropathy slightly improved (strength 4/5) and continued physical rehabilitation. The maintenance treatment continued with PD and AZA; however, the status deteriorated in the following six months with frequent infections and the patient death.

The 8th patient received three doses of ceftriaxone, which was followed by three months of nausea and vomiting (N/V), fatigue, and flank pain. The patient was treated with PD for six months, where the kidney function recovered (sCr = 1.06 mg/dL) and remained stable for three years of follow-up.

Meanwhile, the 9th patient presented with three months of reduced appetite and weight, nocturia, fatigue, and gradual kidney function deterioration. The patient was diagnosed with kidney-limited sarcoidosis when a kidney biopsy showed non-necrotizing granulomas and excluded all other disorders including autoimmune, malignancies, TB, and pulmonary sarcoidosis. The patient received PD for 1 year, where sCr returned to 1.7 mg/dL. The follow-up continued for three years, when the patient was still on a maintenance dose of PD without recurrences.

## Discussion

The prevalence of ATIN differs between countries. It seems to be more common in developing countries, where the prevalence is 7.3% in Morocco, 9.8% in Saudi Arabia, and 8% in Pakistan compared to 2.4-4% in the US and 1-4.7% in different registries from Europe [2]. Also, ATIN seems to be increasing over time, where a Spanish registry (from 1994 to 2009) showed an increase of ATIN from 3.6% in the first 4 years of the registry to 10.5% in the last 4 years [2]. This may reflect the rise in ATIN prevalence specifically due to the increase performance of kidney biopsies for the diagnosis of kidney disorders [2].

Here, we reported ATIN in 9 patients from 2018 to 2022 in a single center in Syria. Of note, additional cases were found before or after 2018, unfortunately, follow-up data or kidney biopsies were not complete for most patients, so we restricted the study to these 5 years. Also, one could observe and suggest ATIN as a common diagnosis without performing a kidney biopsy at all, even though we saw that in clinical practice but we excluded unproved or incomplete ATIN cases, so we could not report all the probable ATIN cases. Similar to a study by Taktak et al. [8], which reported ATIN in 19 patients from the Pediatric Nephrology department between April 1999 and April 2014 in Turkey. This increase in the ATIN prevalence in developing countries might reflect the higher expositions to agents (chemicals or contaminants) or infections. Also, in our country, this might reflect the increasing exposition to chemicals and contaminants after the Syrian war in the previous years, which increased poverty and contaminants.

Rifampin (RIF) is one of the standard treatment protocols for tuberculosis (TB) and brucellosis, which is reported as the most common cause of AKI in anti-TB regimens [9]. RIF is mainly caused by AKI induced by ATIN; however, it may also be associated with Fanconi syndrome and AKI induced by hemolytic anemia [10–12]. AKI may result in continuous or intermittent administration of RIF, which is usually presented with fever, gastrointestinal symptoms, and, less frequently, hepatitis [6]. Although, AKI was also described with a single dose of RIF [12].

Allopurinol and 5-aminosalicylates (5-ASA) are also well-known etiologies of DI-ATIN [2]. ATIN induced by allopurinol seems to be more common in chronic kidney disease (CKD)-patients and may be associated with liver dysfunction and Stevens-Johnson syndrome [2, 6]. The drug reaction with eosinophilia and systemic symptoms (DRESS syndrome) can also be associated with ATIN in 10–30% of cases, particularly with allopurinol and antibiotics [2].

This study reported 5 patients (55.5%) with drug-induced ATIN (DI-ATIN), including rifampicin, allopurinol, mesalamine, and two cases with cephalosporins. The most severe case of AKI was induced by the first dose of RIF. This patient presented with anaphylactic shock and developed AKI, acute liver injury, hemolysis, and thrombocytopenia thereafter. Fortunately, acute liver injury was recovered spontaneously two days later. Whereas, kidney biopsy showed ATIN even with this single dose of RIF, which was recovered by GCs treatment.

Additionally, asymptomatic kidney failure was observed in a routine evaluation of the 5th patient, who had started mesalamine for UC in past few months, whereas the biopsy also showed ATIN. This supports the fact, that 5-ASA can present with acute manifestations of hypersensitivity reaction after the starting of 5-ASA or can be slowly progressed asymptomatically to CKD [2]. On the other hand, only the 4th patient with allopurinol-induced ATIN was diagnosed clinically (without kidney biopsy) when symptoms developed gradually a few months after allopurinol administration. This patient did not show a frank or typical presentation of Stevens-Johnson syndrome; however, the itching sensation was prolonged for nearly a year afterward. Thus, the close monitoring of such patients receiving 5-ASA or allopurinol seems a reasonable approach for an early recognition of kidney impairment. Additionally, two cases of cephalosporins induced ATIN were described, one presented with acute manifestations (2nd patient), and the second showed gradual non-specific symptoms for three months (8th patient).

Tubulointerstitial nephritis and uveitis syndrome (TINU) is a rare condition and is estimated from 0.1 to 2% in all age populations [13]. Uveitis commonly occurs after the onset of ATIN; however, it can occur in less than 2 months before or within 12 months after the onset of nephritis [14]. Surprisingly, asymptomatic uveitis was reported in up to 50% of patients with TINU syndrome, which could be masked by GCs treatment for ATIN disease or might occur during GCs tapering or withdrawal [14]. Uveitis often follows a relapsing-remitting course [2]. Previous data reported high rates of remission of uveitis with GCs treatment; however, 10% of cases showed GCs resistance [14]. Additionally, uveitis relapses were documented with steroid tapering and even with GCs therapy. On the other hand, GCs recently showed insufficiency in preventing uveitis recurrences in 50–70% of patients with TINU syndrome [14]. Whereas these uveitis recurrences often recur with more severe inflammation than the initial event [14].

Indeed, the kidney outcomes in TINU has historically been described with favorable outcome compared to uveitis [14]. Kidney relapses are much less common than uveitis relapses, which may persist or relapse even after 10 years [14]. Also, recent data observed that the progression to CKD was described in a considerable proportion of TINU patients at 1 year of follow-up [14]. Based on all these previous points, even if GCs remain the mainstay in TINU treatment, the relapsing-remitting course of uveitis may frequently mandate a second line or GCs-sparing agents such as AZA, cyclosporine, or mycophenolate mofetil [2, 14].

In this study, the patient with TINU syndrome developed anterior uveitis two months after nephritis, when she started GCs tapering. Additionally, uveitis did not respond to GCs reescalation, so AZA was added, which quickly resolved uveitis inflammation. The follow-up for this patient continued for two years with a good response to AZA treatment without kidney or ocular relapses.

The interstitial inflammation associated with ANCA-associated vasculitis (AAV) is often accompanied by glomerular injury or rarely presents as an isolated form [2]. ATIN induced by AAV was reported only in a small number of cases, which was mostly associated with positive MPO-ANCA [2]. To the best of our knowledge, isolated ATIN induced by AAV was only reported in three cases with positive PR3-ANCA (mostly C-ANCA) [15].

The current study included an exciting case of ATIN induced by AAV with positive C-ANCA (the 7th patient). Systemic manifestations included numbness in all limbs, progressive weakness of the lower extremities, arthralgia, livedo reticularis, hearing loss, anuria, and edema. The association of systemic manifestations aligned with isolated ATIN and positive C-ANCA corresponded with the diagnosis of AAV. This case represents the first case in our country and the fourth case worldwide of ATIN induced by AAV with positive C-ANCA.

On the other hand, isolated ATIN associated with AAV tends to exhibit lower levels of sCr, 24-h urine protein, and MPO titers. Thus, it could be treated with less aggressive regimens, usually GCs monotherapy [15]. It is generally recommended, that the standard induction therapy in patients with organ- or life-threatening AAV including the combination of GCs and either cyclophosphamide (CYP) or rituximab [16, 17]. However, some case studies show promising outcomes of rituximab treatment for neurological involvements, CYP is still the mainstay for severe cases [17].

Thus, our choice in this patient with AAV associated with ATIN and severe neurologic involvement was to administer the induction therapy with GCs and CYP. After a month, the patient showed a good response, where sCr returned to 1.3 mg/dL, which continued stable for the next 6 months. The maintenance treatment continued with PD and AZA; unfortunately, the status deteriorated in the following six months and the patient died.

Granulomatous ATIN is a rare histological finding; however, it represents the most typical form of kidney sarcoidosis, estimated in 70–80% of cases [2, 18, 19]. Sarcoidosis has often been linked to secondary glomerulonephritis, most commonly IgA-nephropathy and membranous glomerulonephritis [18]. Kidney sarcoidosis was hardily to estimate due to the rarity of the disease; one large case series described the incidence of 0.18% [2]. Most patients of ATIN induced by sarcoidosis also have extra-renal manifestations; however, some cases describe a kidney-limited form [2]. Kidney function is often preserved with over 85% of patients responding to GCs treatment [2].

The current study described a rare case of ATIN with non-necrotizing granulomas (the 9th patient). Thus, the patient was diagnosed with kidney-limited sarcoidosis without extra-renal manifestations after excluding all other disorders. Kidney function recovered partially with PD treatment (sCr = 1.7 mg/dL) and the patient’s remission continued for three years with a low-dose maintenance PD.

Pigment nephropathy is a kidney injury resulting as a consequence of endogenous toxins including rhabdomyolysis, intravascular hemolysis, and bile pigment nephropathy [4, 20]. Although, hemolysis is the second most common cause of pigment nephropathy, hemoglobin cast nephropathy is a rare etiology of AKI that requires a massive intravascular hemolysis [4, 20, 21]. The kidney injury in this setting is due to direct heme protein toxicity to tubules, decreased renal perfusion, and intratubular cast formation [4, 20]. Additionally, hemoglobin cast nephropathy can rarely mimic other etiologies of AKI including ATIN and thrombotic microangiopathy [20, 21]. S Mahmud et al. [21] reported two cases of hemoglobin cast nephropathy, where one of them represented features of ATIN. Also, R. Sakthirajan et al. [20] reported pigment nephropathy in 46 patients; including 26 patients with rhabdomyolysis and 20 patients with hemolysis, where only two cases showed ATIN.

In this series, a rare case of G6PD patient presented with gallstone pancreatitis that induced intravascular hemolysis and AKI. Hemoglobin cast nephropathy was primarily the suggested mechanism of AKI; however, when the kidney function did not recover by supportive management, a kidney biopsy was performed. The kidney biopsy showed ATIN superimposed on chronic changes without RBCs or hyaline casts. Thus, PD treatment was introduced for 3 months, which induced a rapid kidney function recovery. The suggested pathogenesis in this patient is the direct hemoglobin toxicity to the kidney tubules and interstitium, which is followed by interstitial inflammation and fibrosis [22].

Several laboratory tests have been studied for ATIN diagnosis; however, there are no specific serum tests that confirm the diagnosis of ATIN [2]. Eosinophilia (20–25% of cases), abnormal urinalysis (pyuria, proteinuria, and hematuria), and elevated CRP have been observed in ATIN patients [2, 4]. Eosinophiluria was previously studied for ATIN diagnosis based on histologic data that showed interstitial infiltrations of eosinophils in DI-ATIN [1]. Subsequent studies show that the absence of eosinophils in kidney biopsy doesn’t exclude DI-ATIN and even eosinophiluria is inaccurate for diagnosing ATIN, which could be observed in other kidney disorders such as glomerulonephritis [1, 4]. Ultimately, up to 20% of ATIN cases could present with normal urinary sediment [4].

Also, ESR frequently shows a nonspecific elevation in ATIN [1]. ESR elevation is generally unhelpful in ATIN diagnosis; however, uncontrolled studies showed that ESR elevation is correlated with a higher degree of interstitial inflammation and a lower degree of fibrosis on biopsy [2]. Anemia is commonly described and frequently observed in association with ATIN; however, it is quietly nonspecific and does not increase the probability of ATIN diagnosis [2]. The noninvasive ATIN diagnosis is quite challenging as there are no definitive clinical features or noninvasive tests for diagnosis [1, 4]. Thus, clinicians should maintain a high suspicion for ATIN diagnosis to further perform a kidney biopsy, which is the only definitive utility for diagnosis [4].

Our study showed a high prevalence of anemia (8 patients; 88.8%), ESR elevation (85.7%), microscopic hematuria (in all patients), pyuria (in 4 patients; 44.4%), and proteinuria (77.7%). In our experience, each one of these tests solely is not specific for ATIN diagnosis; however, the combination of AKI with these positive tests (specifically abnormal urine sediment, ESR elevation, and anemia) may suggest the diagnosis of ATIN and support the treatment initiation particularly when kidney biopsy is unable to perform or when the inciting agent (especially drugs) is predictable. Similarly, Taktak A, et al. [8] reported ATIN in 19 pediatric patients, where only five patients were diagnosed by kidney biopsy, and the other 14 patients were diagnosed clinically by a combination of the history of causative agents, symptoms, and laboratory findings.

Histologic data in ATIN predominantly revealed interstitial infiltrations mainly with lymphocytes and monocytes aligned with eosinophils and neutrophils, which favor DI-ATIN and bacterial infection; respectively [1]. The biopsies of this study also corresponded with these histologic data, which mainly showed interstitial infiltrations with lymphocytes and monocytes. Of note, eosinophils were found in one biopsy and neutrophils showed in 4 biopsies (50%); including TINU, antibiotics, mesalamine, and AAV.

The mainstay of ATIN management is the identification and prompt discontinuation of the offending agent, especially drugs [2, 4]. The overall treatment strategies recommend an induction therapy with high doses of intravenous GCs, followed by oral GCs and then gradual tapering [2]. The main controversial issue is the total treatment duration (induction and tapering), which indeed, is mostly guided by local centers’ experience [2]. The total treatment duration differs between centers as it ranges from a minimum of 4–6 weeks or longer for 8–12 weeks and even to 3–6 months [2, 4].

In those with refractory or relapsed ATIN cases or where GCs are contraindicated, clinicians should consider alternative immunosuppressive agents [2]. Unfortunately, the treatment plan for these cases is not well established; however, other immunosuppressive agents such as CYP, mycophenolate, or AZA are mentioned in the literature [2]. Also, the treatment of ATIN secondary to systemic immune disorders is primarily guided by the recommendations of this autoimmune disease [2, 6].

The major limitation of this study is the limited number of patients from a single center, which defends reporting the prevalence of this disease in our country. However, this study reported rare etiologies of ATIN with a long-term follow-up that enrich the medical literature for managing this disease.

## Conclusion

ATIN seems to be a relatively common cause of AKI in developing countries, reflecting an increased exposition to chemicals and contaminants. Among DI-ATIN, the first dose of rifampin (RIF) induced severe ATIN aligned with liver injury, hemolysis, and thrombocytopenia. Also, mesalamine and allopurinol induced gradual kidney failure a few months after the drug initiation, which supports the need for frequent monitoring of such patients. Additionally, the refractory uveitis in patients with TINU syndrome resolved quickly with AZA after presenting during GCs tapering and not responding to GCs reescalation. Among the rarest cases in this study, one reported kidney-limited sarcoidosis, hemolysis- induced ATIN, and the latter with isolated ATIN induced by AAV with positive C-ANCA, which represents the first case in our country and the fourth case worldwide.

Thus, clinicians should maintain a high suspicion for ATIN diagnosis to further perform a kidney biopsy. Additionally, the combination of AKI with positive tests (especially abnormal urine sediment, ESR elevation, and anemia) could suggest the diagnosis of ATIN and further support the treatment initiation, particularly when kidney biopsy is unable to be performed or when the inciting agent (especially drugs) is predictable.

## Data Availability

No datasets were generated or analysed during the current study.
